# Impacts of Maternal Diet and Alcohol Consumption during Pregnancy on Maternal and Infant Gut Microbiota

**DOI:** 10.3390/biom11030369

**Published:** 2021-03-01

**Authors:** Ying Wang, Tianqu Xie, Yinyin Wu, Yanqun Liu, Zhijie Zou, Jinbing Bai

**Affiliations:** 1 Tongji Hospital, Tongji Medical College, Huazhong University of Science and Technology, 1095 Jiefang Avenue, Wuhan 430030, China; wangying9398@163.com; 2 Wuhan University School of Health Sciences, Wuhan University, 169 Donghu Road, Wuhan 430071, China; 2015302280014@whu.edu.cn (T.X.); 2019283050068@whu.edu.cn (Y.W.); zouzhijie@whu.edu.cn (Z.Z.); 3 Nell Hodgson Woodruff School of Nursing, Emory University, 1520 Clifton Road, Atlanta, GA 30322, USA; jinbing.bai@emory.edu

**Keywords:** gut microbiota, infant, alcohol consumption, maternal diet, pregnancy

## Abstract

(1) Background: Maternal diet and alcohol consumption can influence both maternal and infant’s gut microbiota. These relationships are still not examined in the Chinese population. The purpose of this study was to explore the effect of alcohol consumption and maternal diet during pregnancy on maternal and infant’s gut microbiota. (2) Methods: Twenty-nine mother-child dyads were enrolled in central China. Fecal samples of mothers during late pregnancy and of newborns within 48 h were collected. The V3–V4 regions of 16S rRNA sequences were analyzed. A self-administrated questionnaire about simple diet frequency in the past week was completed by mothers before childbirth. The demographic information was finished by mothers at 24 h after childbirth. (3) Results: Among these 29 mothers, 10 mothers reported alcohol consumption during pregnancy. The PCoA (β-diversity) showed significant difference in maternal gut microbiota between the alcohol consumption group vs. the non-alcohol consumption group (abund-Jaccard, r = 0.2, *p* = 0.006). The same phenomenon was observed in newborns (unweighted-UniFrac full tree, r = 0.174, *p* = 0.031). Maternal alcohol consumption frequency showed positive associations with maternal *Phascolarctobacterium* (*p* = 0.032) and *Blautia* (*p* = 0.019); maternal *Faecalibacterium* (*p* = 0.013) was negatively correlated with frequency of alcohol consumption. As for newborns, a positive relationship showed between *Megamonas* (*p* = 0.035) and newborns with maternal alcohol consumption. The diet was not associated with both maternal and infant’s gut microbiota. (4) Conclusions: Maternal alcohol consumption during pregnancy influenced the gut microbiota on both mothers and the newborns. Future research is needed to explore these relationships in a lager birth cohort. Understanding the long-term effect of alcohol consumption on maternal and newborns’ gut microbiota is needed.

## 1. Introduction

A variety of endogenous and exogenous factors can impact the gut microbiome [1,2]. The Human Microbiome Project (HMP) [3] and the American Gut Project [4] are two initiatives that focus on studying the characteristics of the human microbiome that are associated with both healthy and diseased humans [5]. According to their work, a series of factors can contribute to changes of the human gut microbiome such as demographic factors (e.g., sex, age, ethnicity, geography) [6] and health history (e.g., use of antibiotics) [7]. Importantly, lifestyles, including diet and alcohol consumption are primary factors that influence the development of the gut microbiota in early life [8]. Studies have suggested the existence of mother to child gut microbiota transmission during the neonatal period [9,10] and diet is another most important factor in modifying the composition and diversity of human gut microbiota other than geography and season changes [11]. The impact of both short-term and long-term intakes of different dietary components on changes of gut microbiota have been demonstrated [6,12]. Long-term habitual diet primarily refers diet patterns such as the Mediterranean diet and Western diet. Studies found that the Western diet was associated with high abundance of *Bacteroides* and *Firmicutes* while a plant-based habitual diet showed an increase of beneficial *Bifidobacteria* [6,13]. Additionally, recent epidemiological evidence in a large scale global population reported that dietary patterns can significantly affect gut microbiota composition, richness, and diversity [6,14,15]. Specific food composition-based interventions among certain human populations (e.g., obesity, diabetes, fatty liver, and elevated cholesterol) and animal models suggested that even short-term dietary consumption could significantly affect the composition of gut microbiota [14,16,17].

Besides the effect of diet consumption on gut microbiota, maternal prenatal diet could influence the abundance of infant’s gut microbiota. In an American trial, maternal vitamin D supplementation during pregnancy was associated with lower abundance of *Clostridium difficile* and *Bifidobacterium* but higher abundance of *Bacteroides fragilis* in infants aged 3–6 months [18]. In a New Hampshire birth cohort study, higher abundance of *Streptococcus* and *Clostridium* of infants aged 6 weeks was associated with maternal fruit intake during pregnancy [19]. Another birth-cohort study in Spain [20] showed that maternal fat intake was positively related with neonatal *Firmicute* members and negatively associated with proteins and fiber intake. Similarly, one animal study about Japanese macaque [21] demonstrated that maternal high-fat intake could decrease infants’ gut microbiota diversity at the age of 12 months. Compared with Western countries, Chinese people prefer vegetables and fruits and have different eating habits [22,23]. Until now, the effect of maternal Chinese diet during pregnancy on gut microbiota of newborn is rarely studied, and that from the first feces is needed to be proved urgently.

Women are more likely to drink alcohol during stressful and difficult situations [24,25]. A large number of women who have an unintended pregnancy may consume alcohol prior to the confirmation of pregnancy [26]. Evidence showed that pregnant women have drinking behaviors despite repeated warnings against alcohol consumption during pregnancy [27]. Maternal alcohol consumption has negative consequences on fetal and infant health, including fetal alcohol spectrum disorder [26], sudden infant death syndrome [28], and abnormalities [29]. Association between changes in the gut microbiota and alcohol consumption have been investigated extensively in the last few decades. Sufficient evidence suggested that both acute and chronic exposure to alcohol may lead to specific shift of the microbiota composition [30]. Bull-Otterson et al. [31] found that alcohol leads to a decreased abundance in *Firmicutes* and an increased number of *Actinobaceria* and *Proteobacteria* at phylum level. Evidence in humans also suggested that chronic alcohol consumption caused a decreased *Bacteroidetes* but increased *Proteobacteria*, as well as increased permeability of gastrointestinal tract [32]. However, changes of the gut microbiota in offspring born into alcohol consumption mothers during pregnancy are rarely investigated.

Both alcohol consumption and diet seem to have significant effects on the composition of the human gut microbiota [13,33,34]. However, whether alcohol consumption and diet habits during pregnancy were associated with the gut microbiota in mothers and newborns needs to be further investigated. Therefore, a population-based birth cohort study was performed to assess the influence of maternal alcohol consumption and diet during pregnancy on the gut microbiota of mothers and newborns. The results will confirm the relationship between maternal alcohol consumption, diet habits, and gut microbiota in both mothers and its offspring, which will provide a new angle to improve infants’ health.

## 2. Materials and Methods

Between March 2017 and July 2017, 29 women during late pregnancy were recruited from an obstetric outpatient clinic in a tertiary hospital in central China (Table 1). The inclusion criteria were: (1) women in late pregnancy (≥28 gestational weeks) who planned to deliver a baby in the hospital; and (2) the habitual residence is Wuhan, Hubei Province, China. The exclusion criteria included: pregnant women (1) with complications (e.g., gestational diabetes, hypertensive disorders); (2) receiving antibiotic treatment during pregnancy; or (3) with cognitive impairment.

### 2.1. Data Collection

After admission, a self-designed questionnaire was used to evaluate the 7-day frequency and structure of maternal diet prior to delivery, as well as alcohol consumption during pregnancy. As recommended by the Chinese Society of Nutrition [35], eight common domains of foods for Chinese women during pregnancy were collected, including meat, eggs, fish, vegetables, fruit, milk, nut, and soybean productions. We assessed the diet frequency which involved every day, 5/6 day a week, 3/4 day a week, 1/2 day a week, and never. Alcohol consumption was measured by consumption frequency, categorized as never, rarely, some of the time, and most of the time. Maternal demographic characteristics were collected in the hospital based on maternal self-report at 24 h after childbirth. Fecal samples from women at late pregnancy and their infants after birth within 48 h were collected in the hospital based on the Human Microbiome Project (HMP) protocol [21]. For sequencing and microbial composition analysis, we used Silva (SSU123) 16S rRNA database and the average sequencing depth was 50,000 reads/samples with a minimum of 30,000 reads. Our previous study [36] had elaborated details on DNA extraction, amplification of polymerase chain reaction (PCR), and Illumina MiSeq sequencing.

### 2.2. Statistical Methods

Before the analysis, data of genera relative abundance were transformed into the centered log ratio (CLR). Maternal alcohol consumption was further combined into the following: without alcohol consumption (n = 19) vs. with alcohol consumption (n = 10). For maternal diet, we translated the frequency data into numerical variables, which included: every day = 5, 5/6 day a week = 4, 3/4 day a week = 3, 1/2 day a week = 2, never = 1.

The difference of α-diversity (Shannon index) in alcohol consumption was tested by Student’s t-test at the operational taxonomic unit (OTU) level. To explore β-diversity of alcohol consumption in mothers and infants, principal coordinates analysis (PCoA) was conducted based on abund-Jaccard and unweighted-Unifrac full tree distance matrix and calculated using OTU information from each sample. Analysis of similarities (ANOSIM) was used to check whether these differences were significant. Community heatmap was used to visualize microbiota composition of the two alcohol consumption groups.

The heatmap was used to display the relationships among maternal alcohol consumption, diet frequency, and dominant bacterial communities (genus relative abundance ≥ 1%, based on Spearman correlation test). After adjusting confounders (e.g., gender [male vs. female], delivery mode [natural vs. C-section]), multivariate analysis of general liner model (GLM) was used to analyze significant factors on the mother and infant’s gut microbiota. We set α < 0.05 as the significance level. All the analyses were adjusted using the Benjamini–Hochberg false discovery rate (FDR, < 0.05) in multiple hypothesis tests. These analyses were performed using SPSS version 21 (IBM, Chicago, IL, USA) and R software [37].

## 3. Results

### 3.1. Taxonomies of the Gut Microbiota

**Figure 1:**
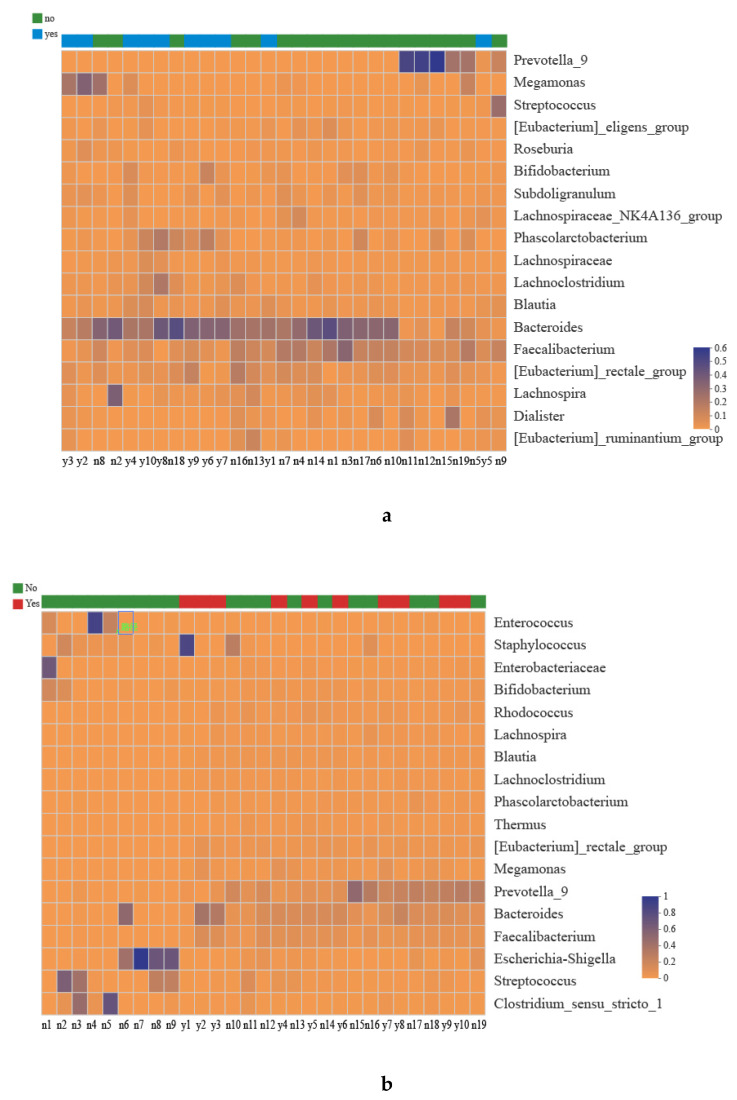
Distribution of microbial community for alcohol consumption group (Yes) vs. without alcohol consumption group (No) at genus level were visualized by community heatmap (≥ 1% relative abundance). The abundance of different samples reflects through color changes: (**a**) indicates maternal gut microbiota composition; (**b**) indicates newborns’ gut microbiota composition.

The values of Good’s coverage were 99.7% in mothers and 99.6% in newborns. In total, 733 OTUs were acquired in maternal fecal samples. We found 98 families and 259 genera were revealed from 29 maternal samples. Among infants, 1480 OTUs were revealed from 29 newborns’ meconium (i.e., the first feces), with 270 families and 579 genera profiled. Figure 1 shows taxonomic compositions of the dominant bacteria (relative abundance > 1%) at genus level. Based on the cutoff point of 1%, 18 genera were identified. The most abundant genera included *Bacteroides* (24.33%), *Faecalibacterium* (10.48%), *Prevotella-9* (7.77%), [*Eubacterium*]_*rectale*_*group* (4.89%), *Phascolarctobacterium* (3.79%), and *Megamonas* (3.71%) for mothers. Eighteen genera were identified in newborns and *Prevotella*_9 (11.74%), *Bacteroides* (11.21%), *Escherichia*-*Shigella* (10.92%), *Streptococcus* (6.78%), *Staphylococcus* (5.58%), and *Clostridium sensu stricto1* (5.11%) were primarily identified.

### 3.2. Microbial Diversity

**Figure 2:**
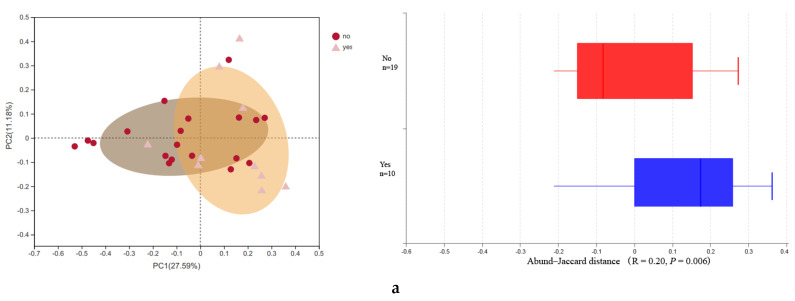

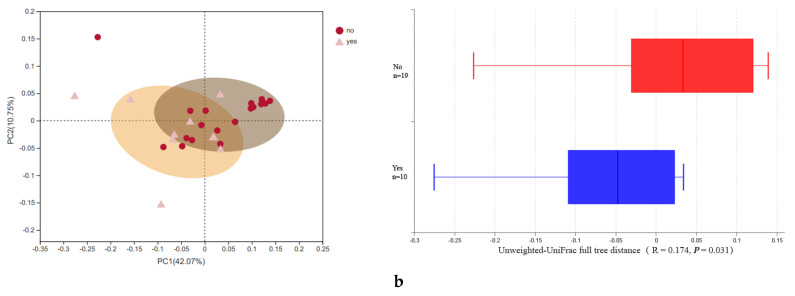
Principal co-ordinates analysis (PCoA) was conducted to display gut microbiota structure difference of the two alcohol consumption status onOTUevel (distance matrix: mothers: abund-Jaccard; infants: unweighted-UniFrac full tree). The statistics of analysis of similarities (ANOSIM) are R and p. If R is closer to 1, it means that the difference between groups is greater than the difference within groups. The Box-plot represented the distribution of different groups of samples on the PC1 axis. (**a**) Indicates the maternal gut microbiota structure difference between with alcohol consumption and without alcohol consumption group; (**b**) indicates newborns’ gut microbiota structure born into mothers with different alcohol consumption.

A significant difference in α-diversity was found between alcohol consumption and without alcohol consumption groups in mothers (*p* = 0.02) and in infants (*p* = 0.03). Mothers with alcohol consumption showed a higher diversity compared with those without alcohol consumption during pregnancy, and similar results had been identified in newborns (Table 1). The β-diversity through PCoA shows that the bacterial structure was separated based on maternal alcohol consumption (Abund–Jaccard, r = 0.2, *p* = 0.006) (Figure 2a). For newborns, the first two principal component scores, accounting for 52.82% of the total variations (unweighted-UniFrac full tree, r = 0.174, *p* = 0.031) suggested that alcohol consumption was one of the important factors in the change of microbiota composition structure (Figure 2b).

### 3.3. Associations between the Maternal Alcohol Consumption, Diet and Gut Microbiota Changes in Mothers and Infants

**Figure 3:**
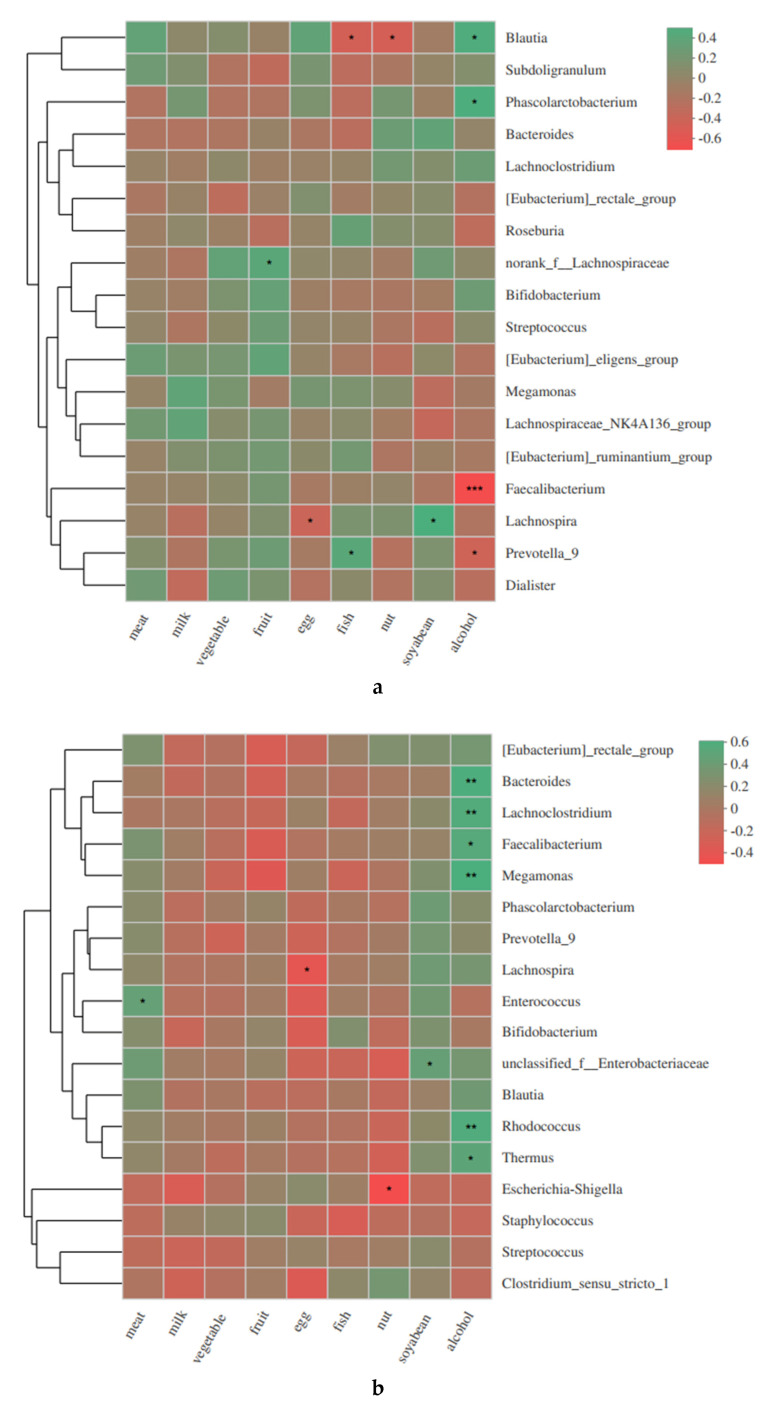
Relationship heatmap was performed to show the association between maternal/newborns’ bacterial abundance and maternal diet intake frequency as well as alcohol consumption before delivery. The coefficient appeared color-coded based on under (red) or over-representation (green) in the analysis (* 0.01 < *p* ≤ 0.05, ** 0.001 < *p* ≤ 0.01, *** *p* ≤ 0.001). (**a**) Indicates relationships among maternal gut microbiota with diet and alcohol consumption; (**b**) indicates relationships among newborns’ gut microbiota with maternal diet and alcohol consumption.

The heatmap shows relationships between gut microbiota changes and maternal diet and alcohol consumption (Figure 3). Significant relationships existed among maternal gut microbiota with intake frequency of fruit (Lachnospiraceae: r = 0.369, *p* = 0.048), egg(Lachnospira: r = −0.373, *p* = 0.046), fish(Blautia: r = −0.443, *p* = 0.016; Prevotella_9: r = 0.379, *p* = 0.042), nut(Blautia: r = −0.448, *p* = 0.015), and soyabean products (Lachnospira: r = −0.443, *p* = 0.011). Moreover, maternal alcohol consumption during pregnancy had a strong relationship with maternal gut microbiota, including *Faecalibacterium* (r = −0.659, *p* = 0.001), *Prevotella_9* (r = −0.407, *p* = 0.029), *Phascolarctobacterium* (r = 0.451, *p* = 0.014), and *Blautia* (r = 0.442, *p* = 0.016). Infants born in mothers who have had alcohol consumption during pregnancy revealed a similar impact on *Bacteroides* (r = 0.512, *p* = 0.005), *Faecalibacterium* (r = 0.460, *p* = 0.012), *Megamonas* (r = 0.521, *p* = 0.004), *Rhodococcus* (r = 0.495, *p* = 0.006), *Lachnoclostridium* (r = 0.483, *p* = 0.008), and *Thermus* (r = 0.426, *p* = 0.021). Maternal dietary intake of meat (Enterococcus: r = 0.376, *p* = 0.044), eggs (Lachnospira: r = −0.374, *p* = 0.046), nut (Escherichia-Shigella: r = −0.454, *p* = 0.013) and soybean products (Enterobacteriaceae: r = −0.373, *p* = 0.046) during pregnancy were related with their offspring’s gut microbiota.

To further investigate the effects of maternal diet and alcohol consumption on maternal and infant’s gut microbiota, GLM was performed to control possible confounders. Results showed that maternal alcohol consumption frequency was significantly related with maternal gut microbiota, including *Phascolarctobacterium* (β = 1771.10, *p* = 0.034, adjust *p* = 0.032) and *Blautia* (β = 538.60, *p* = 0.020, adjust *p* = 0.019). *Faecalibacterium* (β = −3055.7, *p* = 0.001, adjust *p* = 0.013) were negatively correlated with frequency of alcohol consumption. A positive relationship between *Megamonas* in infants and maternal alcohol consumption frequency (β = 1066.53, *p* = 0.030, adjust *p* = 0.035). The α-diversity was not associated with alcohol consumption both in mothers and infants.

## 4. Discussion

In this study, alcohol consumption has significant impact on both maternal and offspring’s gut microbiota. Mothers, who had alcohol consumption during pregnancy, had a significantly lower abundance in *Faecalibacterium* genus and a higher abundance in *Phascolarctobacterium*, *Blautia* genera compared with those without alcohol drinking groups. These findings were consistent with previous studies [31,32,38,39]. In a study with a pregnant animal model, reduced *Bacillus* was observed in pregnant mice exposure in ethanol [40]. Interestingly, plenty of research studies have proved the transmission of gut microbiota between mothers and offspring in utero. Studies [39,41,42] involving maternal diet, gestational weight gain (GWG), and antibiotic treatment have reinforced that point of view. Accordingly, several hypotheses [42] were proposed about transmission routes (e.g., vagina, placenta, amniotic fluid, cord blood, and fetal membranes) which means that maternal prenatal factors can alter the initial newborns’ gut microbiota. In the study, for newborns with mothers who have consumed alcohol during pregnancy, we observed higher abundance in *Megamonas* genus. The over-growth of *Megamonas* in newborns further indicated that alcohol consumption during pregnancy might affect offspring’s gut microbiota colonization in early life [43].

The negative effect of maternal alcohol consumption during pregnancy is undoubted, both on mothers and newborns. In this study, mothers with alcohol consumption during pregnancy had a lower-level abundance of *Faecalibacterium*. *Faecalibacterium* was shown to involved in intestinal anti-inflammatory [44] and play a role in preventing alcohol-induced gastrointestinal and extra-intestinal diseases [45,46] by blocking nuclear factor κB (NF-κB) [47]. High intestinal permeability (IP) was positively related to the abundance of *Blautia* genus [48]. Additionally, the prevalence of *Phascolarctobacterium* was believed to associated with hepatic diseases including nonalcoholic steatohepatitis (NASH) [49] and hepatocellular carcinoma (HCC) [50]. In addition, *Blautia* was known to be elevated in people with depressive symptoms [51]. Furthermore, a number of studies in animals about the effect of alcohol on the gut microbiota also showed that alcohol consumption caused a decreased abundance of *Firmicutes* and increased numbers of *Verrucomicrobia* and *Bacteroidetes*, and *Firmicutes* have been confirmed, which was associated with anti-inflammatory activity in human body [38]. Canesso et al. [52] transplanted fecal samples of mice fed with alcohol into germ-free mice, and there was an increase of gut and hepatic inflammation which suggested that gut microbiota can mediate alcohol proinflammatory effect. Besides, in this study, *Megamonas* was abundant in infants exposed to alcohol consumption mothers. Additionally, increased *Megamonas* was proved to be associated with major depressive disorder [53]. Moreover, prenatal alcohol espouse was linked to changes in newborns’ cognitive and behavioral development including depression, mood diseases, and autism [54,55]. In utero, MRI also demonstrated [56] that maternal ethanol exposure in gestation could perturb brain development associated motor control. According to Kakiyama’s study, infants born into mothers who have alcohol consumption during pregnancy may result in hepatic damage, along with an increased number of Bacteroides [30]. Further, long-effect on offspring existed even low-level alcohol exposure during pregnancy [55]. In summary, alcohol consumption mediated mothers’ gut microbiota composition and passed the effect on infants. However, their gut microbiota composition showed different changes. The intergenerational transmission of *Faecalibacterium*, *Phascolarctobacterium*, *Blautia*, and *Megamonas* genera were not found in this study. Other cohort study [57] has pointed out that excessive *Lachnospiraceae* genus transmission was observed in overweight mothers and overweight infants aged 1 and 3 years old. Nevertheless, plenty of factors were associated with the transmission process, including infants’ gestational age, delivery mode, antibiotic usage, and the operating room microbes [58]. It may be the possible cause that different gut microbiota change patterns occurring in mothers and infants in this study. Besides that, further long-term clinical studies were strongly recommended, taking factors into account such as alcohol categories, specific levels of alcohol consumption during pregnancy. It would help investigate roles of alcohol consumption in maternal and infants’ gut microbiota more accurately.

In our study, we explored the effect of meat, eggs, fish, vegetables, fruit, milk, nut, and soybean productions on maternal and infants’ gut microbiota. Although some maternal diet was associated with gut microbiota in mothers and infants, they were not significantly controlling confounder factors. However, studies have proved that [59] eggs intake was correlated with *Lachnospiraceae* and *Blautia*. Soybeans and grains as the main source of polyphenols including isoflavones and flavones were the primary dietary influencing factors on *Lactobacillus*, *Bifidobacterium*, and *Enterococcus avium* [60,61,62,63,64]. It also found [59] that pulses intake was negatively related to *clostridium*. In addition, the changes were translated significantly into gut microbiota of infants [65,66,67]. A previous study showed that high fat or oral probiotic supplement can drive gut microbiota changes in offspring [65]. Maternal *Lactobacillus rhamnosus* GG (LGG) consumption increased the abundance of *Bifidobacteria* colonization in infants [68]. The abundance of *Bacteroides* decreased in newborns exposure to maternal high-fat diet during pregnancy [69]. However, different duration and quantity of diet components intake may lead to various results in gut microbiota [70], based on the microbe–host co-metabolism [71]. Likewise, 35% of bacterial OTUs were related to eating time and frequency. Human gut microbiota changed throughout the day with a decreased concentration of butyrate, acetate, and propionate [72]. It seems to explain different findings in the relationships between diet and gut microbiota.

## 5. Conclusions

In conclusion, these results showed that maternal alcohol consumption during pregnancy induced changes of the gut microbiota in mothers and their offspring. In the area of maternal and infant health care, this study has provided compelling evidence that limiting maternal alcohol consumption is of benefit to both mothers and infants. Among limitations of our study, the dietary and alcohol assessment may be subjected to memory bias. Specific effects of different degrees and different kinds of alcohol consumption on the maternal and infant gut microbiota are still unclear. Future research should lay great stress on food/alcohol quantity or proportion and minimize bias as possible. Besides, a limited participant size results in less representative of the study. Thus, it is in urgent need to develop a large birth cohort and explore the long-term impact of alcohol consumption and diet on infant and mother’s gut microbiota.

## Figures and Tables

**Table 1:** Characteristics of maternal and infant birth cohort (N = 29).

Variable		Maternal Alcohol Consumption		*p* Value
		**Yes (10, 34.38%)**	**No (19, 65.52%)**	
Maternal age		29.8 ± 2.04	30.9 ± 4.15	0.44
Pre-BMI		20.77 ± 3.02	21.15 ± 2.63	0.73
GWG		15.95 ± 1.63	15.53 ± 4.52	0.82
Gestational week		40.13 ± 1.08	39.55 ± 4.15	0.15
Mode of delivery	C-section	7 (24.14%)	8 (27.59%)	0.16
	Vaginal delivery	3 (10.34%)	11 (37.93%)	
Infant gender	Male	5 (17.24%)	5 (17.24%)	0.24
	Female	5 (17.24%)	14 (48.28%)	
Newborns’ alpha diversity	Shannon	4.01 ± 1.07	2.62 ± 1.70	0.03 *
Maternal alpha diversity	Shannon	3.66 ± 0.50	3.19 ± 0.49	0.02 *

Values listed are total for the variable (percent of total value n) or means ± SD. Across rows (*) indicate means that are significantly different (*p* < 0.05). Pre-BMI: pre-pregnancy body mass index; GWG: gestational weight gain.

## Data Availability

The data presented in this study are openly available in Sequence Read Archive: SRA:SRP155310; BioProject: PRJNA482931.
